# AtTrxh3, a Thioredoxin, Is Identified as an Abscisic AcidBinding Protein in *Arabidopsis thaliana*

**DOI:** 10.3390/molecules27010161

**Published:** 2021-12-28

**Authors:** Tomoaki Anabuki, Keisuke Ohashi, Taichi E. Takasuka, Hideyuki Matsuura, Kosaku Takahashi

**Affiliations:** 1 Division of Fundamental Agriscience Research, Research Faculty of Agriculture, Hokkaido University, Kita 9 Nishi 9, Kita-ku, Sapporo 060-8589, Japan; bukio0119@gmail.com (T.A.); takasuka@cen.agr.hokudai.ac.jp (T.E.T.); matsuura@agr.hokudai.ac.jp (H.M.); 2 Graduate School of Global Food Resources, Hokkaido University, Kita 9 Nishi 9, Kita-ku, Sapporo 060-0809, Japan; keiroy124@eis.hokudai.ac.jp; 3 Department of Nutritional Science, Faculty of Applied Bioscience, Tokyo University of Agriculture, 1-1-1 Sakuragaoka, Setagaya-ku, Tokyo 165-8502, Japan

**Keywords:** abscisic acid, affinity chromatography, *Arabidopsis thaliana*, click chemistry, target protein, thioredoxin

## Abstract

Abscisic acid (ABA, **1**) is a plant hormone that regulates various plant physiological processes such as seed developing and stress responses. The ABA signaling system has been elucidated; binding of ABA with PYL proteins triggers ABA signaling. We have previously reported a new method to isolate a protein targeted with a bioactive small molecule using a biotin linker with alkyne and amino groups, a protein cross-linker, and a bioactive small molecule with an azido group (azido probe). This method was used to identify the unknown ABA binding protein of *Arabidopsis thaliana*. As a result, AtTrxh3, a thioredoxin, was isolated as an ABA binding protein. Our developed method can be applied to the identification of binding proteins of bioactive compounds.

## 1. Introduction

**Figure 1:**
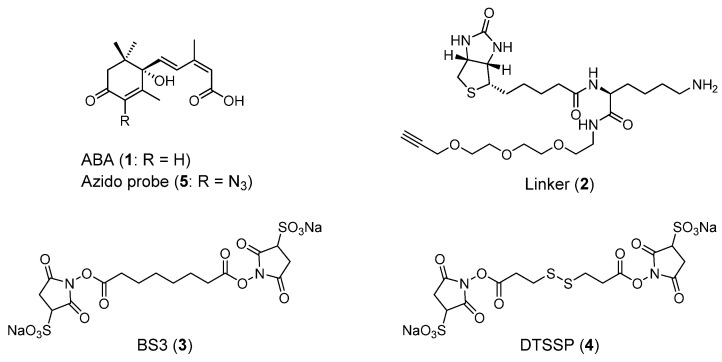
Compounds in this study. BS3: bis(sulfosuccinimidyl)suberate disodium salt; DTSSP: dithiobis(sulfosuccinimidyl propionate) disodium salt.

Abscisic acid (ABA, **1**, Figure 1) is a plant hormone that regulates plant developmental processes and stress responses [1,2]. ABA induces embryo-specific genes in developing seed, which causes accumulation of proteins and lipids, desiccation tolerance, and germination inhibition. Moreover, environmental stresses such as drought, cold, and high salinity elevate ABA concentrations, causing an accumulation of the mRNA of stress-responsive genes, leading to stress tolerance. The identification of ABA receptors is a topic that has been under intensive study. In 2009, soluble ABA receptors, called PYL proteins, were identified [1,2]. PYLs indirectly regulate the activity of SNF1-related kinases (SnRK2s) that phosphorylate many stress-activated targets in response to ABA. Core components of ABA signaling are PYLs, Mg^2+^-dependent protein phosphatase 2C (PP2C), and SnRK2s. In non-stress conditions, PP2C directly inhibits phosphorylation of SnRK2s. When environmental stresses or developmental cues increase ABA concentrations, ABA binds to PYLs and changes the conformation of PYLs, allowing the binding of PYLs with PP2C. PP2C bound to the complex of ABA and PYLs abolishes the activity of phosphorylation. Therefore, activated SnRK2s trigger protein phosphorylation, leading to the activation of transcriptional and downstream factors involved in ABA signaling.

On the other hand, affinity-based methods to identify ABA binding proteins have been tried. A biotinylated analogue of ABA, which was fused with benzene ring, identified ABA-8′-hydroxylase [3], a mitochondrial adenine nucleotide transporter [4], and ribulose-1,5-bisphosphate carboxylase/oxygenase (Rubisco) [5] as ABA binding proteins. In these studies, a biotin group was introduced in ABA derivatives as a partial structure and reduced plant hormonal activity as compared to ABA. The direct introduction of large functional groups into original bioactive compounds often reduces biological activities significantly.

**Figure 2:**
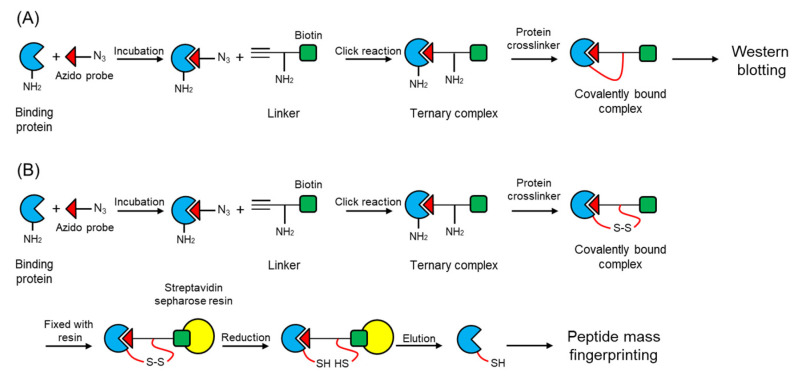
Illustration of the methods used for identifying a target protein using a probe with an azido group. (**A**) Chemical labeling; (**B**) Identification of a target protein.This complex is unable to be dissociated under physiological conditions. A target protein in the stable covalently bound ternary complex was detected by Western blotting using streptavidin conjugated with horseradish peroxidase (Figure 2A). Moreover, after the click reaction, the ternary complex was covalently fixed with a protein cross-linker with an intramolecular disulfide bond such as dithiobis(sulfosuccinimidyl propionate) disodium salt (DTSSP, **4**, Figure 1 and Figure 2B). The covalently bound ternary complex was immobilized with a streptavidin resin. Non-specifically associated proteins with the affinity matrix were washed thoroughly, and the disulfide bond was then cleaved by a reducing agent, resulting in the release of the target protein from the affinity matrix (Figure 2B). The eluted proteins were analyzed by peptide mass fingerprinting. The method described above succeeded in identifying a recombinant AtPYL2 from a protein extract of *E. coli* that overexpressed AtPYL2 [7].

We have developed new methods for chemical labeling and identification of a protein targeted by a bioactive small molecule [6,7]. The outline of the methods is depicted in Figure 2. For chemical labeling, after the target protein was associated with a bioactive compound with an azido group (azido probe), a linker with a terminal alkyne, an amino group of lysine, and a biotin (**2**, Figure 1 and Figure 2A) were bound to the complex of target protein by click reaction. The ternary complex was covalently fixed with a protein cross-linker such as bis(sulfosuccinimidyl)suberate disodium salt (BS3, **3**, Figure 1 and Figure 2A). 

## 2. Results and Discussion

### 2.1. Isolation of an ABA Binding Protein

**Figure 3:**
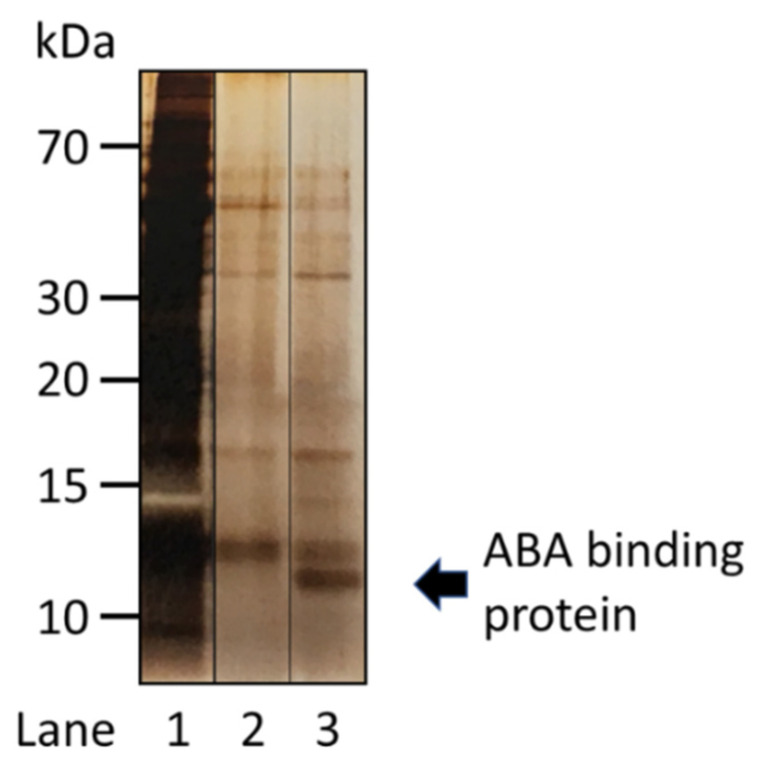
Isolation of an ABA binding protein in *A.thaliana*. After the incubation of a protein extract of *A. thaliana* with **5**, bio-orthogonal coupling of **2** to **5** was performed, and the ternary complex was connected by DTSSP **4**. The ternary complex was bound with a streptavidin sepharose. The proteins in each fraction were analyzed by SDS-PAGE and then visualized by silver staining. Lane 1: pass through; lane 2: wash (50 mM phosphate buffer (pH 7.8) with 100 mM NaCl and 2 M urea); lane 3: elution (50 mM phosphate buffer (pH 7.8) with 100 mM NaCl, 2 M urea and 100 mM DTT).

The developed method for chemical labeling and identification of a target protein in previous studies was applied for identification of an ABA binding protein in *A. thaliana*. At first, we identified AtPYLs by the method described above. PP2C was considered to prevent the approach of linker **2** to an azido probe of ABA (**5**, Figure 1) bound with AtPYLs; therefore, an *A. thaliana* mutant with a disrupted PP2C gene (SALK_038866C) was used. Azido probe **5** was incubated with a protein extract of the *A. thaliana* mutant, and linker **2** was then conjugated with azido probe **5** via Huisgen cycloaddition [8,9,10]. An ABA binding protein and a conjugate of linker **2** and azido probe **5** were cross-linked through amino groups in linker **2** and an ABA binding protein using DTSSP (**4**), a protein cross-linker containing a disulfide bond. The complex of the ABA binding protein, linker **2**, and azido probe **5** was immobilized to a streptavidin sepharose. After washing with a buffer containing 2 M urea, an ABA binding protein was eluted with a buffer containing dithiothreitol (DTT). SDS-PAGE analysis showed that several protein bands were present in the washed fraction and the eluted fraction. Comparing bands detected in both fractions, the significant difference was that the band, for which the molecular mass was approximately 12 kDa, was clearly detected in the DTT-eluted fraction (Figure 3).

### 2.2. Identification of the Isolated Protein

**Figure 4:**
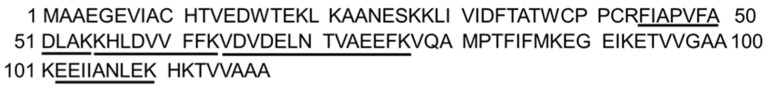
The amino acid sequence of AtTrxh3 and the identified peptides by peptide mass fingerprinting. ABA binding protein was separated by the affinity chromatography described above. AtTrxh3 (NP_199112) was identified by peptide mass fingerprinting. The tryptic peptide fragments identified by nano LC-MS/MS are underlined.

After excising the band from the gel, the protein was digested by trypsin, with subsequent analysis by nano LC-MS/MS [11]. The data of MS (Table S1) and SDS-PAGE suggested that AtTrxh3 (NP_199112), a thioredoxin, was an ABA binding protein (Figure 3 and Figure 4). The molecular mass of AtTrxh3 was 13.1 kDa, which was slightly higher than that observed in Figure 3. A part of N- or C-terminal region in AtTrxh3 was possibly removed during the isolation steps. AtPYL was not detected in this study, as expected. It is possible that AtPYLs were more difficult to detect due to much lower abundance as compared to AtTrxh3.

Thioredoxins are a group of small redox proteins that are ubiquitously present and are crucial for various biological processes in living organisms [12]. They are necessary for various biological processes such as redox signaling. Thioredoxins are oxidoreductases containing two reactive cysteines in a conserved motif CXXC. The two cysteines are critical for the ability of thioredoxin to reduce other proteins and are recycled by NADPH-dependent thioredoxin reductase.

### 2.3. Binding of a Recombinant AtTrxh3 Produced with ABA

**Figure 5:**
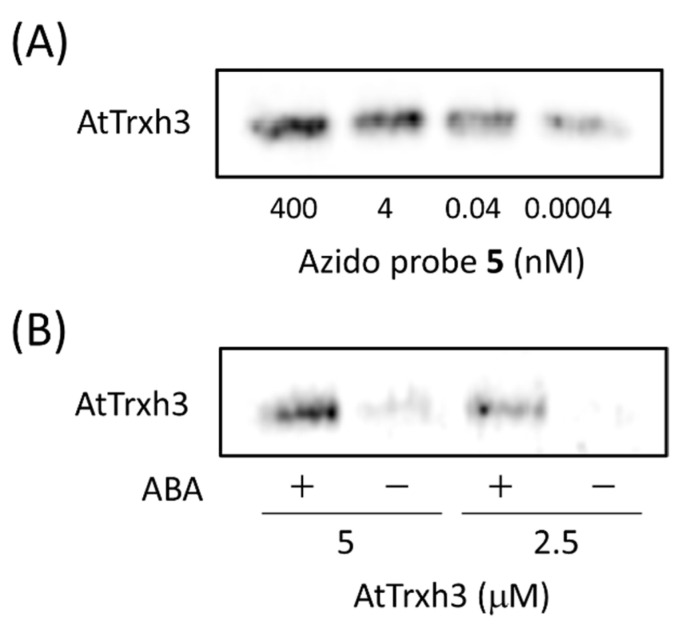
Evaluation of the dose-dependent effects of the concentrations of azido probe **5** and AtTrxh3 on signal intensity derived from AtTrxh3 in Western blotting. (**A**) The concentration of azido probe **5** was varied from 0.0004 to 400 nM. The concentration of linker **2** and DTSSP **4** was 40 µM, and AtTrxh3 concentration was 5.0 μM. (**B**) The concentration of AtTrxh3 was varied from 2.5 to 5.0 μM. The concentration of azido probe **5** was 40 µM, and the concentrations of linker **2** and DTSSP **4** were 40 µM. Plus and minus signs respectively indicate the presence and absence of a 50-fold excess amount of ABA.

To confirm that ABA binds with AtTrxh3, a recombinant AtTrxh3 produced in *E. coli* (Figure S1) was used for Western blotting. We then investigated whether the intensity of the chemiluminescent signal varied with azido probe **5** (Figure 5A). In the concentration range of azido probe **5** from 0.0004 to 400 nM, a dose-dependent increase in the chemiluminescent signal of AtTrxh3 was observed. The results indicated that the intensity of the chemiluminescent signal dose-dependently increased until 4 nM of azido probe **5**, at which point it reached a plateau and the further addition of azido probe **5** did not enhance the chemiluminescent signal. Accordingly, chemiluminescent signal intensity arising from AtTrxh3 was also enhanced in a concentration-dependent manner of azido probe **5**. A dose-dependent increase in the chemiluminescent signal was observed at a concentration range of AtTrxh3 until 5.0 μM (Figure 5B). The data revealed that intensity of chemiluminescent signal derived from AtTrxh3 increased in a dose-dependent manner with AtTrxh3. Moreover, excess amounts of ABA significantly reduced the chemiluminescent signal intensity. Therefore, ABA was clearly shown to bind with AtTrxh3.

Previous report showed that our developed method was effective for isolating recombinant AtPYL2, a receptor of ABA [7]. As far as we know, the binding of ABA with AtTrxh3 has not yet been reported. This study indicated that the method was effective not only as a model experiment but also as a practical method for identifying a binding protein of a bioactive compound in a plant extract. Our developed method using a substrate-derived bioactive small molecule, a biotin linker with alkyne and amino groups, and a protein cross-linker can be applicable for target protein identification.

AtTrxh3 has been reported to show the activities of thioredoxin and chaperonin [13]. Thioredoxin activity and chaperonin activity are respectively involved in reduction of reaction oxygen species and refolding proteins. *A. thaliana* mutant overexpressing *AtTrxh3* gene was reported to be tolerant to heat shock stress. Accordingly, these activities are related to stress adaptation in *A. thaliana* [13]. On the results of such previous studies, we examined whether ABA affects AtTrxh3 activities such as thioredoxin and chaperonin. Unfortunately, ABA did not influence activities relating to the thioredoxin and chaperonin of AtTrxh3 in vitro (data not shown). Thioredoxins have been shown to be involved in adaptations for heat shock and cold stresses [13]. Cytosolic h-type thioredoxins have been shown to act as key regulators of seed germination [12,14]. Moreover, ABA induces Trxh accumulation in seedlings of wheat (*Triticum durum*) [15]. Considering that ABA also influences these physiological reactions, some biological phenomena caused by AtTrxh3 in vivo may be induced via the binding of ABA with AtTrxh3. Further study is needed on how the binding of ABA with AtTrxh3 influences AtTrxh3-mediated physiological reaction.

## 3. Materials and Methods

### 3.1. Synthesis of Linker **2** and Azido Probe **5**

Linker **2** and azido probe **5** were synthesized according to the methods of Anabuki et al. [6].

### 3.2. Affinity Chromatography

The seeds of an *Arabidopsis* mutant with a disrupted PP2C gene (SALK_038866C) were obtained from the Arabidopsis Biological Resource Center, Columbus, OH, USA. The *Arabidopsis* mutant was grown in a growth chamber under white luminescent light (8 h light/16 h dark) at 22 °C for 5 weeks. The shoots (0.8 g) were frozen by liquid nitrogen and powdered by a mortar and pestle. The powdered sample was dissolved in 10 mL of 50 mM phosphate buffer (pH 7.8, 100 mM NaCl) and stirred at 4 °C for 8 h. The suspension was centrifuged at 5000 rpm and the obtained supernatant was used as a protein extract. Azido probe **5** (40 μM) was dissolved in 1 mL of a protein extract (protein concentration, 1.0 mg/mL, 50 mM phosphate buffer, pH 7.8, 100 mM NaCl) and then preincubated for 1 h at 4 °C. Huisgen cycloaddition was initiated via the addition of 40 μM of linker **2** and 0.8 μM of CuI saturated aqueous solution, and the mixture was incubated for 1 h. Then, 40 μM of DTSSP **4** (Dojindo Laboratory, Kumamoto, Japan, 1 mM), a protein cross-linker, was added to the resultant supernatant, which was then incubated for an additional 1 h. The protein solution was applied to a Streptavidin HP SpinTrap column (GE Healthcare, Little Chalfont, UK) along with 400 μL of 50 mM phosphate buffer (pH 7.8) containing 100 mM NaCl and 2 M urea. The same wash procedure was performed 3 times. The target protein was eluted with 400 μL of 50 mM phosphate buffer (pH 7.8) containing 100 mM NaCl, 2 M urea and 100 mM DTT. The same elution procedure was performed twice. The proteins in each fraction were analyzed by 15% SDS-PAGE and were stained by silver staining.

### 3.3. Proteome Analyses

The band of the target protein was excised from the gel after SDS-PAGE. The gel was decolorized using destaining reagents for silver staining (Nacalai Tesque, Kyoto, Japan). Thus, 500 µL of reductive alkylation buffer 1 (10 mM DTT, 100 mM NH_4_HCO_3_, 0.3% EDTA, pH 8.5) was added to the gel and incubated for 15 min at 25 °C. After removal of the solvent, 500 µL of reductive alkylation buffer 2 (100 mM acrylamide, 100 mM NH_4_HCO_3_, 0.3% ethylenediaminetetraacetic acid, pH 8.5) was treated with the gel for 15 min at 25 °C. After reductive alkylation buffer, the gel was washed using 100 mM NH_4_HCO_3_ aqueous solution. Trypsin digestion was performed by adding proteomic-grade trypsin (Apro Science, Tokushima, Japan) for 12 h at 37 °C. The peptides were extracted by 50% acetonitrile containing 0.1 % formic acid and purified by a ZipTip-C18 column (Merck, Kenilworth, NJ, USA). Mass spectra were obtained by using an Easy nLC1000 liquid chromatography system with a Q-exactive Plus Orbitrap mass spectrometer (Thermo Fisher Scientific, Waltham, MA, USA) and Xcalibur software (ver. 3.1, Thermo Fisher Scientific, Waltham, MA, USA). The peptides were separated on a C18 capillary tip column (NTCC-360/75-3-125, Nikkyo Techno, Japan) by a linear gradient from 5 to 30 % over 120 min in 0.1 % formic acid in acetonitrile. Full scan mass spectra were obtained in the Orbitrap with a scan range of 300.0 to 2000.0 *m/z* with a resolution of 70,000. Proteins were identified from the acquired MS/MS spectra using Proteome Discoverer 2.1 (Thermo Fisher Scientific, Waltham, MA, USA). The peptide mass tolerance was set at 10 ppm, and the fragment mass tolerance was set at 0.8 Dalton. The peptide charge was set at +2, +3 and +4. The accuracy and sensitivity of peptide identification were optimized using the automatic decoy and percolator functions of the Proteome Discoverer software.

### 3.4. Production of Recombinant AtTrxh3

A single colony of *E. coli* transformed with a pET23 vector containing *AtTrxh3* gene (AT5G42980) was incubated in 1 L of LB medium containing 100 μg/mL of ampicillin at 37 °C. After the culture had grown to a cell density of OD_600_ = 0.6, AtTrxh3 synthesis was induced by the addition of IPTG at 0.2 mM. After further incubation at 25 °C for 3 h, the cells were collected by centrifugation at 5000× *g* for 10 min and then resuspended with 50 mM sodium phosphate buffer (pH 7.8) with 100 mM NaCl and disrupted by ultrasonication. The cell debris was removed by centrifugation at 12,000 rpm for 10 min. The resultant supernatant was loaded on a Ni-sepharose column chromatography to purify a recombinant AtTrxh3 in a standard method.

### 3.5. Detection of AtTrxh3 by Western Blotting

A complex of AtTrxh3, linker **2**, and azido probe **5** was analyzed by SDS-PAGE. The gel was equilibrated with blotting buffer (25 mM Tris-HCl buffer, pH 8.3, 192 mM glycine, 10% MeOH) for 5 min. A PVDF membrane (Fujifilm Wako, Osaka, Japan), which had been treated with MeOH and water, was also equilibrated with the blotting buffer. The protein was transferred from the gel onto the PVDF membrane over a 1 h period using a Trans-blot semi-dry SD cell (Bio-Rad Laboratories, Hercules, CA, USA). The electric current was set at 0.8 mA/cm^2^. For blocking, the membrane was incubated in 5 % non-fat dry milk in PBS-T buffer (80 mM Na_2_HPO_4_, 20 mM NaH_2_PO_4_, 100 mM NaCl and 0.1% Tween 20) for 1 h at 25 °C and then washed twice using PBS-T buffer. After blocking, the membrane was incubated in PBS-T buffer containing streptavidin conjugated to horseradish peroxidase (0.2 µL/mL, Thermo Fisher Scientific, Waltham, MA, USA). After washing the membrane, the membrane was soaked in Western blotting detection reagents (Fujifilm Wako, Osaka, Japan) at room temperature for 5 min. The chemiluminescence derived from the ternary complex was detected using a Lumi-Vision Pro 400EX fluorescence imager (AISIN, Kariya, Japan).

### 3.6. Evaluation of the Dose-Dependent Effects of the Concentrations of Azido Probe **5** and AtTrxh3

The protein concentration of AtTrxh3 was varied from 2.5 to 5 μM. Variable amounts of azido probe **5** (from 0.0004 to 400 nM) were added to the reaction mixture. The procedure for AtTrxh3 detection was performed as described above.

### 3.7. Activities of Thioredoxin of AtTrxh3

The thioredoxin reductase reaction was conducted in 50 mM phosphate buffer (pH 7.8) containing 10 μM AtTrxh3, 10 mM 5,5′-dithiobis(2-nitrobenzoic acid) (DTNB), and 0.4 mM NADPH in 50 mM phosphate buffer (pH 7.8), and was incubated at 25 °C for 1 h. The thioredoxin reductase activity was measured by absorbance at 412 nm in the presence or absence of 0.5 mM ABA.

### 3.8. Activity of Chaperonin of AtTrxh3

The activity of chaperonin of AtTrxh3 was examined according to the method of Park et al. [13].

## Data Availability

The data presented in this study are available on request from the corresponding author.

## Supplementary Materials

The following are available online, Figure S1: SDS-PAGE analysis of a recombinant AtTrxh3, Table S1: nano LC-MS/MS data of tryptic peptide fragments of AtTrxh3.

## Sample Availability

Samples of the compounds are available from the authors.
