# Supplementary material for: AtTrxh3, a Thioredoxin, Is Identified as an Abscisic AcidBinding Protein in Arabidopsis thaliana
Source: Molecules. 2021 Dec 28;27(1):161. doi: 10.3390/molecules27010161 (PMC8746859; doi:10.3390/molecules27010161)
Supplement: Supplementary file 1 [file molecules-27-00161-s001.zip › molecules-1481923-supplementary.pdf]

Supplementary Materials

AtTrxh3, a Thioredoxin, Is Identified as an Abscisic Acid Binding Protein in *Arabidopsis thaliana*

Tomoaki Anabuki [1], Keisuke Ohashi [2], Taichi E. Takasuka [1,2], Hideyuki Matsuura [1] and Kosaku Takahashi [3,*]

[1] Division of Fundamental Agriscience Research, Research Faculty of Agriculture, Hokkaido University, Kita 9 Nishi 9, Kita-ku, Sapporo, 060-8589, Japan; bukio0119@gmail.com (T.A.), takasuka@cen.agr.hokudai.ac.jp (T.E.T.), matsuura@agr.hokudai.ac.jp (H.M.)

[2] Graduate School of Global Food Resources, Hokkaido University, Kita 9 Nishi 9, Kita-ku, Sapporo, 060-0809, Japan; keiroy124@eis.hokudai.ac.jp

[3] Department of Nutritional Science, Faculty of Applied Bioscience, Tokyo University of Agriculture, 1-1-1 Sakuragaoka, Setagaya-ku, Tokyo, 165-8502, Japan

* Correspondence: kt207119@nodai.ac.jp; Tel.: +81-3-5477-2679.

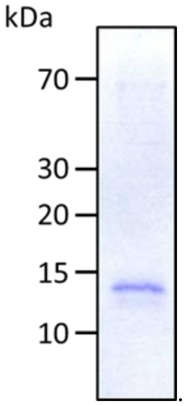

Figure S1. SDS-PAGE analysis of a recombinant AtTrxh3. A recombinant AtTrxh3 synthesized in *E. coli* was analyzed by SDS-PAGE and then stained by CBB (coomassie brilliant blue).

Table S1. nano LC-MS/MS data of tryptic peptide fragments of AtTrxh3.

Annotated Sequence	Retention Time (min)	Charge	*m/z* (Da)	MH^+ (Da)	Delta *m/z* (Da)
FIAPVFADLAK	88.946311	2	596.34246	1191.67766	0.000219082
KHLDVVFFK	64.484702	3	378.22189	1132.65112	-0.000071569
VDVD-ELNTVAEEFK	86.678969	2	804.39398	1607.78068	0.000389554
EEIIANLEK	64.040562	2	529.79083	1058.57438	0.000786105
